# Local ocular surface findings in COVID-19 patients without ocular symptoms

**DOI:** 10.5935/0004-2749.2021-0419

**Published:** 2023

**Authors:** Gözde Aksoy Aydemir, Burcin Pehlivanoglu, Emre Aydemir, Burak Oren, Halil Ibrahim Atesoglu, Yasin Sakir Goker

**Affiliations:** 1 Ophthalmology Department, Adiyaman University Training and Research Hospital, Adiyaman, Turkey; 2 Department of Pathology, Faculty of Medicine, Dokuz Eylul University, Izmir, Turkey; 3 Ophthalmology Department, Ulucanlar Eye Training and Research Hospital, Ankara, Turkey

**Keywords:** Coronavirus infectious, COVID-19, SARS-CoV-2, Ocular manifestations, Tears, Infecções por coronavirus, COVID-19, SARS-CoV-2, Manifestações oculares, Lágrimas

## Abstract

**Purpose:**

To investigate subjective ocular symptoms and objectively measure tear secretion in patients with a confirmed diagnosis of coronavirus disease-2019 (COVID-19).

**Methods:**

In this prospective cross-sectional study, 24 patients who had survived COVID-19 infection and 27 healthy controls were enrolled. Conjunctival impression cytology, the Schirmer test, tear-film break-up time, corneal staining scores were applied to all the participants.

**Results:**

No significant difference was noted with regard to the gender and mean age between the two groups (p=0.484 and p=0.599, respectively). The conjunctival impression cytology analysis revealed that the density of the goblet cells was decreased, while the counts of lymphocytes and neutrophils were increased in the COVID-19 group patients when compared with ethe control group patients. When the Nelson classification was applied to the conjunctival impression cytology samples, 25% of the COVID-19 group patients and 14.8% of the control group patients exhibited changes consistent with ≥grade 2. The mean tear-film break-up time, Schirmer test, and corneal staining score results were determined to differ between the COVID-19 and control groups (p=0.02, p<0.001, and p=0.003, respectively).

**Conclusions:**

The present study revealed the pathological conjunctival alterations of patients with a confirmed diagnosis of COVID-19, indicating the possibility of the occurrence of pathological ocular surface alterations to even at the end of COVID-19 infection, without the occurrence of any significant clinical ocular manifestations.

## Abstract (pt)

**Objetivo:**

Investigar sintomas oculares subjetivos e medir a secreção lacrimal objetivamente em pacientes com diagnóstico confirmado da doença coronavírus 2019 (COVID-19).

**Métodos:**

Vinte e quatro pacientes que sobreviveram à infecção pela COVID-19 e 27 controles saudáveis foram incluídos neste estudo transversal prospectivo. Citologia de impressão da conjuntiva, teste de Schirmer, tempo de separação do filme lacrimal, pontuações de coloração da córnea foram aplicados a todos os participantes.

**Resultados:**

Concluiu-se que não houve diferença significativa em relação ao gênero e idade média entre os dois grupos (p=0,484 e p=0,599, respectivamente). A análise dos resultados da citologia de impressão da conjuntiva revelou que a densidade das células do cálice diminuiu, enquanto os linfócitos e neutrófilos aumentaram nos pacientes do grupo COVID-19 quando comparados com os do grupo controle. Quando a classificação de Nelson foi aplicada às amostras de citologia de impressão da conjuntiva, determinou-se que 25% dos pacientes do grupo COVID-19 e 14,8% dos pacientes do grupo controle apresentaram alterações consistentes com grau 2 ou superior. O tempo médio de separação do filme lacrimal, teste de Schirmer e os resultados das pontuações de coloração da córnea foram determinados, diferindo entre o grupo COVID-19 e o grupo controle (p=0,02, p<0,001, and p=0,003, respectivamente).

**Conclusões:**

As análises realizadas neste estudo revelaram as alterações conjuntivais patológicas de pacientes com diagnóstico confirmado de COVID-19 e mostraram que é possível que alterações patológicas da superfície ocular ocorram mesmo no final da infecção pela COVID-19, sem a ocorrência de manifestações oculares clínicas significativas.

## INTRODUCTION

On December 31, 2019, cases of unknown pneumonia began to be reported in the hospitals of Wuhan, China’s Hubei Province^(1)^. After 2 weeks, it was reported that the infectious agent causing this severe acute respiratory failure was identified to be a novel enveloped RNA beta-coronavirus, which was named severe acute respiratory syndrome-coronavirus 2 (SARS-CoV-2) in the Coronaviridae family^(2)^. Gradually, the cases spread to the entire world. The World Health Organization declared that this epidemic had evolved into a pandemic on March 11, 2020^(2)^.

The main route of transmission for this novel SARS-­CoV-2 is through close contact with infected individuals through respiratory droplets expelled during coughing or sneezing. The upper respiratory tract and the mucosa of the conjunctiva are connected. It is believed that the conjunctiva can be easily affected in individuals infected with SARS-CoV-2, as it may be a possible route of transmission for the virus^(3)^. In addition, both the conjunctiva and the epithelium in the cornea possess angiotensin-con­verting enzyme-2 (ACE-2) receptors. Hence, they may play a significant role in assisting and easing the entry of the virus into the host membrane cells^(4)^.

In past studies, SARS-CoV-2 was identified in the conjunctival swabs of the infected individuals^(5-7)^. While patients may present to the clinic with conjunctivitis as the first symptom, the presence of the virus in the tears and conjunctival secretions has been reported in patients without conjunctivitis^(8)^. In addition, a recent study that evaluated ocular surface inflammation detected pathological changes in COVID-19 patients without the presence of any ocular involvement^(9)^.

However, to the best of our knowledge, there is no detailed information available on the medium/long-term effects of COVID-19 on the ocular surface epithelium. Hence, the present study objective comprised an investigation using the tear-film break-up time and conjunctival impression cytologic analyses, and the Schirmer test and ocular surface disease index (OSDI) questionnaire results of patients with the confirmed diagnosis of COVID-19 and a healthy control group to achieve a much clearer understanding of the subtle ocular involvement of this disease.

## METHODS

### Case selection

This study, which was been designed with a cross-sectional and prospective structure, was conducted at our hospital in the Department of Ophthalmology. The approval for this study protocol was provided by the institutional board of the local ethics committee, and the study was designed such that it adhered to the ethical principles contained within the Declaration of Helsinki (approval number: 304/2020, approval date: 25/06/2020). This study included 32 patients who had survived COVID-19 infection as well as 36 healthy controls between 1 and 30 July 2020. Informed written consent forms were obtained from all participants in both the study groups before their admission into the study. A total of 8 patients group and 9 healthy controls with an insufficient impression of cytology samples were excluded from this study.

The 24 study subjects were determined to have contracted COVID-19 through the identification of SARS-CoV-2 in their respiratory specimens through nucleic acid testing in conjunction with a real-time reverse transcriptase-polymerase chain reaction assay, and they tested negative after the treatment. The ocular examinations were performed 14-30 days after the onset of their COVID-19 symptoms following the confirmation of a negative reverse transcriptase-polymerase chain reaction test result. During the COVID-19 treatment, only patients who undertook antiviral and/or anticoagulant treatment were included. All the patients included in this study exhibited mild COVID-19 symptoms and none required hospitalization or ventilation. In this study, the ocular surface changes in outpatients who did not require hospital treatment were examined. The eye complaints of each patient were retrospectively questioned before and during their illness. Signs of secretion, burning, stinging, itching, and hyperemia were also recorded. Patients who did not report any ocular symptoms during or after COVID-19 infection were included in this study.

The control group was composed of individuals who were healthy and had been matched for age and sex and showed negative results on their reverse transcriptase-polymerase chain reaction test for at least 3 days. Moreover, they reported no prior history of diagnosis of COVID-19 infection. The participants in the control group were selected from healthy patients who had applied for a standard eye examination at the ophthalmology clinic and had not been previously diagnosed with any ocular or systemic diseases. The examination performed in this study was conducted on a randomly selected eye of each of the participants.

The subjects bearing the following medical conditions were excluded from the study: a history of abusing chronic ocular drugs; wearing contact lenses; using topical medications; undergone laser treatment or ocular surgery in the previous year; having secondary ocular and/or systemic diseases with the manifestation of dry eyes; having any opacification in the media; history of the infectious or noninfectious corneal or conjunctival disease; corneal trauma; a history of any ocular surgery that may cause ocular surface disorders; a history of any systemic disease associated with ocular surface disorders; as well as redness, itching, secretion, discharge, blepharitis, conjunctivitis, or episcleritis. In addition, all patients who expressed inadequate willingness to cooperate in their examinations were also excluded from the study. Finally, a total of 24 patients who were confirmed to have been infected with COVID-19 and who met the inclusion criteria were incorporated into the study.

In line with the recommendations set forth by the Dry Eye Workshop Group, the tests and measurements were conducted in the following sequence: first, the measurement of the tear-film break-up time, followed by the corneal staining score, and finally the Schirmer test^(10)^. The OSDI questionnaire was applied before the ocular tests. Beginning with the conjunctival impression cytologic sampling, the tear-film break-up time, corneal staining score, and Schirmer tests were performed at 15-min intervals. All ocular measurements in the present research were conducted by the same researcher.

Examinations of all patients were conducted under the same conditions. All examinations were conducted in the morning to standardize the tests and avoid the possibility of diurnal variations. All assessments were performed in a room with dim lighting and regulated airflow, temperature, and humidity to avoid the occurrence of ocular surface stress. Any measurements that required the use of a slit lamp were conducted in a room that had darkened lighting, and they were all performed by the same physician using the same slit lamp to minimize bias.

### OSDI scoring

The OSDI questionnaire-developed by the Outcome Research Group (Allergan Inc., Irvine, CA, USA)-contains 12 items and is used to rapidly and reliably assess dry eye symptoms. It is also available in the Turkish language version, which has been determined to be both valid and reliable^(11)^. All the patients in the study completed the questionnaire on their own, without any assistance from the clinician, before having their ophthalmic examinations. Each item on the questionnaire was graded based on a 5-point scale, in which 0 = patient never experienced any symptoms; 1 = sometimes experienced some symptoms; 2 = experienced symptoms half of the time; 3 = experienced symptoms most of the time, and 4 = always experienced the symptoms. The total OSDI score was calculated using the following formula: OSDI score = (the sum of all scores for all questions answered × 100)/(the total number of questions answered × 4)^(11)^. An OSDI score ≥13 was considered to indicate the presence of dry eye disease.

### Tear-film break-up time test

The measurement of the tear-film break-up time was performed by using a fluorescein dye solution. After the solution was applied to the patients, they were asked to blink thrice to ensure that the fluorescein dye was adequately mixed with the tear film. The time interval between the last blink and the observation of the first dark spot on the cornea was measured consecutively, in seconds, using a stopwatch. Three consecutive measurements were made and averaged, and this average value was accepted as the tear-film break-up time. A diagnosis of dry eye disease was made if the tear-film break-up time was <10 s.

### Corneal staining score

The ocular surface was examined by fluorescein staining^(12)^. Next, 1% preservative-free fluorescein dye was introduced into the conjunctival sac. The corneal fluorescein stain was assessed 3 min following the introduction of the fluorescein, for which a slit lamp, which omitted a cobalt blue light, was used to observe the cornea. For the cornea, a total of 5 areas were considered for measurement. Evaluation of the area that exhibited corneal staining was performed with a score ranging from 0 (= no staining) to 3 (= widespread loss of the epithelium). The results depicted a total corneal staining score of 0-15. A corneal staining score ≥3 was accepted as abnormal^(13)^.

### Schirmer test

To evaluate the basal and reflex tear secretions, the Schirmer test was performed without the induction of anesthesia. In this test, the number of tears produced within a 5-min period was recorded using a filter paper strip. Briefly, a filter paper strip was placed at the intersection of the middle and lateral third of the lower eyelid. Ambient lighting was used to conduct the test. The patients were asked to look in a forward direction and blink normally throughout the test. The wetness acquired by the filter paper in 5 min was recorded in millimeters. The Schirmer test could estimate the tear fluid secretion and the tear volume of the patients.

### Conjunctival impression cytology

**Figure 1:**
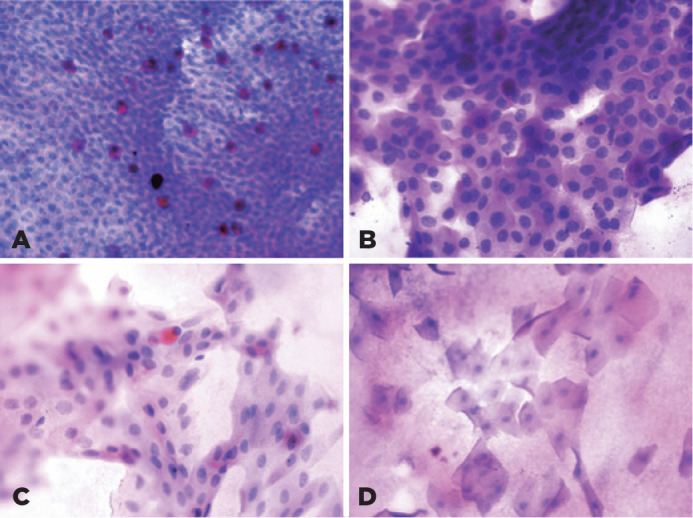
Grading as per the Nelson scoring system. (A) Grade 0, basaloid squamous surface cells with abundant goblet cells. Note the prominent mucin globules within the goblet cells. (B) Grade 1, conjunctival surface epithelial cells with a nucleus: the cytoplasm ratio of 1:2 to 3 with a decrea­sed number of goblet cells. (C) Grade 2 is characterized by the presence of intermediate-like squamous cells (i.e., the cells with a nucleus: cytoplasm ratio of 1:4 to 5) and only a few goblet cells, and (D) Grade 3, superficial-like squamous cell (nucleus: cytoplasm ratio >1:6) without any goblet cells. Periodic acid Schiff stain, x200, x400, x400, and x200, respectively.

The eyes of the patient were topically anesthetized to perform the impression cytology of the conjunctiva. Small disks of cellulose acetate filter paper (MFS; Advantec MFS, Pleasanton, CA, USA; pore size = 0.2 µm) were sliced into approximately 4 × 5-mm pieces, placed on the superior nasal bulbar conjunctiva at a distance of 5 mm from the limbus, gently pressed for 5 s, and subsequently removed. The specimens were placed in a 96% ethanol solution and stained with periodic acid Schiff stain, as described previously^(14)^. Periodic acid Schiff-stained conjunctival impression cytologic specimens were examined blindly by a pathologist (B.P.) and graded according to the Nelson scoring system based on the morphology of the epithelial cells and the number of goblet cells (Figure 1)^(14)^. The number of goblet cells per mm^2^ (approximately 4 high-power fields) was recorded. Moreover, the numbers of neutrophils and lymphocytes were counted in a high-power field.

### Statistical analyses

The analyses of the data in this study were conducted using the IBM SPSS Statistics for Windows 22.0 (IBM Corp., Armonk, NY, USA). Descriptive data were reflected as the mean ± standard deviation, percentage, and frequency distribution. The analysis of the categorical variables was performed using Pearson’s Chi-square and 1-sample Chi-square tests. The normal distribution of the variables was assessed using both analytical methods (i.e., Kolmogorov-Smirnov and Shapiro-Wilk tests) and visual (i.e., probability graphs and histogram). For comparison of the patient and control groups, the independent sample t-test was applied for normally distributed data, while the Mann-Whitney U-test was applied for the non-normally distributed data. To examine any existing relationships among the measured variables, Pearson’s correlation analysis was performed. For all analyses, p<0.05 was considered to indicate statistical significance.

## RESULTS

In the present research, 51 eyes from 51 individuals were included, of whom 24 patients belonged to the COVID-19 group and 27 individuals to the control group. No statistically significant differences were determined between the age and gender of the participants between the 2 groups (p>0.05). All ocular examinations were conducted within 14 to 30 days after the patients had received confirmation of negative reverse transcriptase-polymerase chain reaction results for COVID-19 infection.

The slit lamp bio-microscopy examinations of the conjunctiva and eyelid margins of the participants revealed no coexistent blepharitis or meibomian gland disorders, and no ocular surface fluorescein staining was observed in either of the groups.

The Schirmer test, corneal staining score, and tear-film break-up time values indicated that the differences between the patient and control groups were significant (p<0.001, p=0.003, p=0.02). Moreover, no significant differences were recorded between the patient and control groups in terms of the OSDI scores (p=0.089). The clinical characteristics of the participants in the study and the outcomes of their ocular measurements are depicted in table 1.

**Table 1:** Demographic characteristics of the study participants and their ocular measurement outcomes

	**COVID-19 group** (n=24)	**Control group** (n=27)	**p-value**
Age (year)	55.21 ± 6.90	56.15 ± 6.80	0.599^**^
Gender (female/male)	15/9	13/14	0.484^**^
Tf-BUT	9.45 ± 1.64	10.88 ± 2.45	0.02^*^
Schirmer test	10.08 ± 1.41	14.55 ± 3.52	<0.001^*^
OSDI score	13.0 ± 1.69	12.25 ± 1.34	0.089^*^
Corneal staining score	1.67 ± 1.57	0.56 ± 0.89	0.003^*^

COVID-19= Coronavirus disease 2019; Tf-BUT= Tear-film break-up time; OSDI= Ocular Surface Disease Index; n= the number of eyes, Bold denotes that p<0.05 was statistically significant. ^*^Independent sample t-test. ^**^Chi-square test.

**Figure 2:**
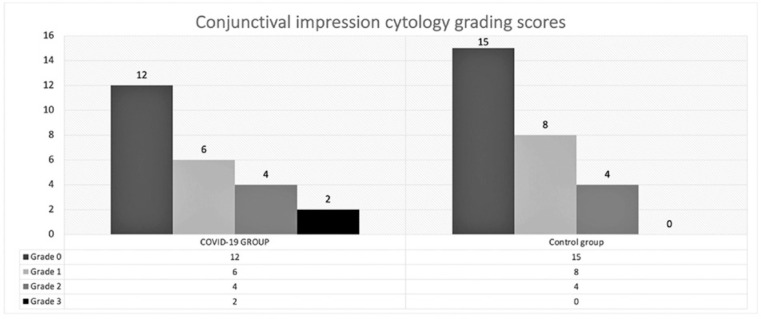
Comparison of the impression cytology grading scores for both groups.

The conjunctival impression cytological analysis revealed a decrease in the number and size of the goblet cells, with larger epithelial cells of polygonal shape; and a decreased ratio of nuclei to the cytoplasm, with a greater degree of basophilic staining in the COVID-19 group relative to that in the control group (Figure 1). The numbers of goblet cells were 108.64 ± 124.81 and 119.70 ± 90.42 in the COVID-19 and control groups, respectively (p=0.721). Similarly, inflammatory cell infiltration was found to be more prominent in the COVID-19 group, albeit without any statistical significance. When the conjunctival impression cytologic results were assessed by the Nelson classification, 25% (n=6) of the samples exhibited changes consistent with those of classification of grade ≥2 in the COVID-19 group. On the other hand, 14.8% (n=4) of the samples exhibited changes consistent with those of the classification of grade ≥2 in the control group (Figure 2). There were no grade 3 cases in the control group. The mean number of the goblet cells significantly decreased by grade (p<0.001). The corneal staining score was significantly higher in the COVID-19 grades 2-3 group when compared to the COVID-19 grades 0-1 and control grades 0-1 groups (p=0.032 and p=0.001); the mean value was 3.17 vs. 1.17 and 0.57, respectively. Schirmer test results were significantly shorter in the COVID-19 group than that in the control group (COVID-19 grades 0-1: 10.05 mm, COVID-19 grades 2-3: 10.16 mm vs. control grades 0-1: 14.3 mm, control grade 2: 16 mm, p<0.05). In the COVID-19 group, the conjunctival impression cytologic grade was significantly negatively correlated with the tear-film break-up time and positively correlated with the corneal staining scores, although the number of the goblet cells was significantly negatively correlated with the corneal staining score alone (Table 2).

**Table 2:** Correlation of the CIC specimens and Schirmer test, Tf-BUT, OSDI, and corneal staining score results in the COVID-19 group

	**Schirmer test**		**Tf-BUT**		**OSDI**		**Corneal staining score**	
	p-value	**r**	**p-value**	**r**	**p-value**	**r**	**p-value**	**r**
Grading scores	0.418	-0.173	**0.027**	-0.451	0.404	-0.178	**<0.001**	0.674
Goblet cells	0.103	0.357	0.442	0.173	0.394	0.191	**0.013**	-0.521
Lymphocyte	0.234	-0.253	0.745	-0.070	0.603	-0.112	0.461	-0.158
Neutrophils	0.495	-0.146	0.835	0.045	0.327	-0.209	0.729	-0.075

COVID-19= Coronavirus disease 2019; CIC= Conjunctival impression cytology; Tf-BUT= Tear-film break-up time; OSDI= Ocular Surface Disease Index Bold denotes that p<0.05 was statistically significant. r= Pearson’s correlation coefficient.

## DISCUSSION

In this case-control prospective research, a higher number of dry eye diseases were recorded in individuals diagnosed with COVID-19 relative to those in the control group. The Schirmer test and tear-film break-up time values were found to be significantly lower, while the corneal staining scores were significantly higher in the COVID-19 group when compared to that in the control group. Inflammation reportedly plays a major role in dry eye disease pathogenesis and active and chronic inflammation in the ocular surface cells^(15,16)^. It has been reported that an increase in the interleukin-1 (IL-1), IL-6, TNF alpha, and IL-17 inflammatory cytokines in the conjunctiva epithelium and cornea is indicative of inflammation occurring on the ocular surface^(17)^. Inflammatory cytokines activate the secretions from the inflammatory cells on the ocular surface. Such inflammatory events result in the apoptotic death of surface epithelial cells, such as goblet cells; this damage to the goblet cells has a direct relation to the effects caused by chronic inflammation^(18)^. Considering that COVID-19 patients who were within their recovery period were included, the lack of notable inflammation in the COVID-19 group compared to that in the control group is not surprising. However, more importantly, more frequent occurrence of dry eye disease and grade 2 and 3 cases in the COVID-19 group suggests that goblet cell recovery following inflammation may take a long time. It has been demonstrated that the host immune response, more specifically the exaggerated release of proinflammatory cytokines, plays a major role in the clinical course of COVID-19^(19,20)^. This proinflammatory microenvironment may also be responsible for the protracted regeneration of the conjunctival goblet cells. Although these findings suggest that dry eye disease may be a complication of COVID-19, the fact that non-hospitalized patients without apparent ocular involvement by the disease were included may lead to an underestimation of dry eye disease as a complication of COVID-19.

In the present study, the OSDI questionnaire-a quick method to evaluate symptoms related to dry eye-was applied to determine the presence of subclinical ocular findings. In their study, Hong et al. administered the OSDI questionnaire via phone calls to 56 patients after discharge^(21)^. The authors reported that the complaints of patients with pre-coronavirus dry eye symptoms increased and that patients without symptoms developed dry eye symptoms after getting infected. Although this finding supports the hypothesis that dry eye disease is a complication of COVID-19, longitudinal studies are warranted to monitor the changes in OSDI during and after the disease up to several months of the recovery period. Significantly lower Schirmer test and tear-film break-up time scores and significantly higher corneal staining scores were recorded in the COVID-19 group, with significant correlations noted among conjunctival impression cytological grading scores, tear-film break-up time, and corneal staining scores. A strong negative correlation was noted between the corneal staining scores and the number of goblet cells. Although these correlations suggested that impression cytology is a sensitive method for identifying dry eye disease in COVID-19, unfortunately, no significant difference was noted between the COVID-19 and control groups in terms of the mean number of goblet cells per mm^2^ and the Nelson grades despite the higher frequency of grade 2 cases in the COVID-19 group and the lack of grade 3 cases in the control group. Although this finding can most likely be attributed to the small number of subjects, it may also reflect the limited diagnostic value of impression cytology in this particular patient group. Previously, it was reported that the Schirmer test and tear-film break-up time decreases significantly despite normal grading scores and goblet cells in non-COVID-19 diseases, implying a higher sensitivity^(22,23)^. Whether this notion also applies to COVID-19 warrants further investigation.

Ocular problems caused by COVID-19 have been demonstrated in several studies^(6,9)^. However, in the studies conducted to date, epidemiological data on the incidence of conjunctivitis have been reported as 0.8-7.9% in patients with COVID-19^(6,7,24,25)^. Wu^(7)^ reported that conjunctival congestion occurs in patients with more severe COVID-19. Bozkurt et al.^(9)^ recorded conjunctival morphological changes caused by COVID-19, which were evaluated through impression cytology, similar to that in the current study. However, unlike in the current study, their conjunctival impression samples were collected 3 h after the reverse transcriptase-polymerase chain reaction analyses of their patients were confirmed to be positive. The authors noted higher grade scores and fewer goblet cells in their patient group relative to those in their control group. While significant differences were recorded between the COVID-19 and control groups in the present study also, ophthalmological examination and conjunctival impression cytologic sampling were performed within 14-30 days of receiving a negative reverse transcriptase-polymerase chain reaction result, that is, in patients who had recovered from COVID-19. Therefore, it is believed that these two studies reflect different aspects of the disease.

There were some limitations to the present study. As the patients were not evaluated at the time of their initial diagnosis, a comparative study design could not be performed. The cross-sectional nature of the study, the small sample group, and the absence of RT-PCR testing for SARS-CoV-2 in these conjunctival samples along with the strict exclusion criteria limited the implications of the study findings. This study included patients who had mild COVID-19 that did not require hospitalization and/or ventilation and who did not describe ocular symptoms and other systematic diseases.

It is therefore opined that the application of the strict inclusion criteria eliminated any medication side-effects and ventilation on ocular examination findings and thus provides a more objective evaluation of the changes related to COVID-19.

In future studies, it is necessary to evaluate patients from the time of their first diagnosis until follow-up. Moreover, examining the virus in the patients’ tears through RT-PCR analysis at the time of the first diagnosis is expected to provide more accurate results.

Our findings indicated a decreased number of goblet cells and increased grading scores of patients diagnosed with COVID-19, not only at the time of diagnosis but also during the subsequent follow-up. Even when their COVID-19 symptoms were mild, the dry eye parameters of the patients changed significantly. This finding suggests the possible presence of ocular surface changes without clinically significant ocular symptoms, not only at the time of diagnosis but also during the subsequent follow-up.
